# What Do We Know About Hybrid Blue ( *Balaenoptera musculus* ) and Fin ( *B. physalus* ) Whales? A Comprehensive Review Across Ocean Basins

**DOI:** 10.1002/ece3.73630

**Published:** 2026-05-05

**Authors:** Christophe Pampoulie, Valerie Chosson, Sverrir Daníel Halldórsson, Trevor A. Branch, Marc Ruiz‐Sagalés, Alex Aguilar, Guðjón M. Sigurðsson

**Affiliations:** ^1^ Marine and Freshwater Research Institute Hafnarfjörður Iceland; ^2^ School of Aquatic and Fishery Sciences University of Washington Seattle Washington USA; ^3^ Institut de Recerca de la Biodiversitat (IRBio) and Departament de Biologia Evolutiva, Ecologia i Ciències Ambientals (BEECA), Facultat de Biologia, Universitat de Barcelona Barcelona Spain; ^4^ Reial Acadèmia de Ciències i Arts de Barcelona (RACAB) Barcelona Spain

**Keywords:** blue whale, fin whale, genetic, hybridisation, morphology, North Atlantic Ocean, Pacific Ocean, Southern Ocean

## Abstract

Hybrids between blue ( *Balaenoptera musculus* ) and fin ( *B. physalus* ) whales have been reported from commercial whaling since the late 1800s. Even while species descriptions were still at their infancy, whalers described whales with intermediate characteristics between blue and fin whales. In more recent decades, hybrid reports have come mainly from sighting surveys or genetic analysis from biopsies or caught individuals. Over the past three decades, hybrids have been confirmed genetically with various methods, which have provided information related to maternal lineages. Furthermore, hybrids captured by recent whaling operations have enabled genetic verification and detailed assessment of their morphological traits. In this review, we synthesise available information on blue–fin whale hybrids across ocean basins, encompassing historical accounts and contemporary observations, including recently documented specimens. We document 46 possible hybrids, of which 17 have been genetically confirmed: 15 from the North Atlantic, one from the Mediterranean, and one from the north‐east Pacific. We also highlight possible bias in current knowledge and recommend the collection of biopsies when atypical whales that exhibit characteristics of both species are encountered.

## Graphical abstract

This review paper summarises the current knowledge on hybrids between blue and fin whales, from historical to contemporary observations, highlighting morphological and genetic identification across the world oceans.

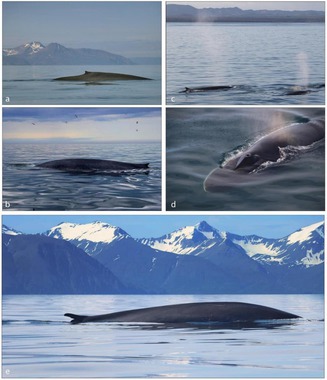

## 1 Introduction

The blue whale ( *Balaenoptera musculus* ) and the fin whale ( *B. physalus* ), the two largest animals on Earth, have been roaming across all oceans for nearly eight million years (Árnason et al. 2018; Westbury et al. 2019). There is a substantial overlap of blue and fin whale distributions and diet that results in ecological niche overlap and potential competition over food resources (Friedlaender et al. 2015; Leroy et al. 2018; García‐Vernet et al. 2021). Both species are believed to undertake long, annual migrations between high‐latitude summer feeding areas and low‐latitude winter breeding areas, although evidence is accumulating on the presence of blue and fin whales at high feeding latitude during the winter (Širović et al. 2004; Simon et al. 2010; Morano et al. 2012; Gunnlaugsson and Víkingsson 2014) and of blue whales staying in temperate and tropical waters year‐round (Reeves et al. 2004; McDonald et al. 2009; Barlow et al. 2018).

Both blue and fin whales were severely depleted across their distribution range during the 20th century (Christensen 2006; Pershing et al. 2010; Branch et al. 2004). The blue whale was driven to the brink of extinction due to modern whaling that started in northern Norway in 1868 with the inventions of vessel motorization, harpoon systems with explosive heads, and improved towing capabilities, then spread globally, notably to the Antarctic in 1904 (Tønnessen and Johnsen 1982). The invention of the stern slipway in the mid‐1920s also spurred pelagic whaling and allowed the hunting of these fast‐swimming whales in open waters anywhere in the world (Tønnessen and Johnsen 1982). Blue whale populations became depleted earlier and more severely than fin whale populations, leading to the cessation of blue whale exploitation in 1954 in the North Atlantic and elsewhere in 1966. However, until 1978, some countries such as Spain, which were not members of the International Whaling Commission (IWC) and therefore not subject to its catch limits, carried out sporadic catches (Aguilar and Borrell 2022). Fin whale exploitation ceased in the Southern Hemisphere in 1976, before the global moratorium on commercial whaling had been approved by the IWC in 1982, coming into effect from the 1985/86 whaling season.

Catch levels varied across ocean basins. In the North Atlantic, approximately 11,000 blue whales and 81,000 fin whales were killed for commercial purposes starting in 1868 (Allison 2024). Globally, around 90% of all catches of these species were in the waters around Antarctic, with global totals of 386,619 blue whales and 886,621 fin whales (Allison 2024).

Because of high levels of catches and only belated protection from whaling, global blue whale population abundance declined from an estimated 340,280 to fewer than 5000 individuals worldwide, and the global population of fin whales decreased from 762,400 to 109,600 individuals (Christensen 2006). Today, the most recent estimates of population from the IUCN Red List of Threatened Species were established in 2018 for both species (Cooke 2018a, 2018b). Blue whales were listed as Endangered under criteria A1abd (the reduction of population size was calculated on a combination of direct observation (a), indices of abundance (b) and exploitation data (d)), with global population estimates of mature individuals ranging from 5000 to 15,000 individuals (Cooke 2018a), while fin whales were globally listed as Vulnerable under criterion A1d (the reduction of population size was only calculated using exploitation data (d)) with increasing trends and a global population size of about 100,000 individuals (Cooke 2018b). Globally, both species fall under criterion A1 which states that: ‘the population reduction was observed, estimated, inferred, or suspected in the past where the causes of the reduction are clearly reversible, understood and have ceased’.

The pronounced disparity in current abundance between these species may influence interspecific interactions, including hybridization (Pampoulie et al. 2021). Indeed, Pampoulie et al. (2021) suggested that the unidirectional hybridisation process observed during their study, that is, that most of the hybrids resulted from the successful mating of a blue whale mother with a fin whale father, could be aligned with the ‘Sexual selection hypothesis for unidirectional hybridization’ (Wirtz 1999). This hypothesis crucially depends on the relative abundance of the respective species involved in the hybridisation process and predicts that females of the rarer species may eventually accept heterospecific males when conspecific mates are scarce (Wirtz 1999).

**FIGURE 1:**
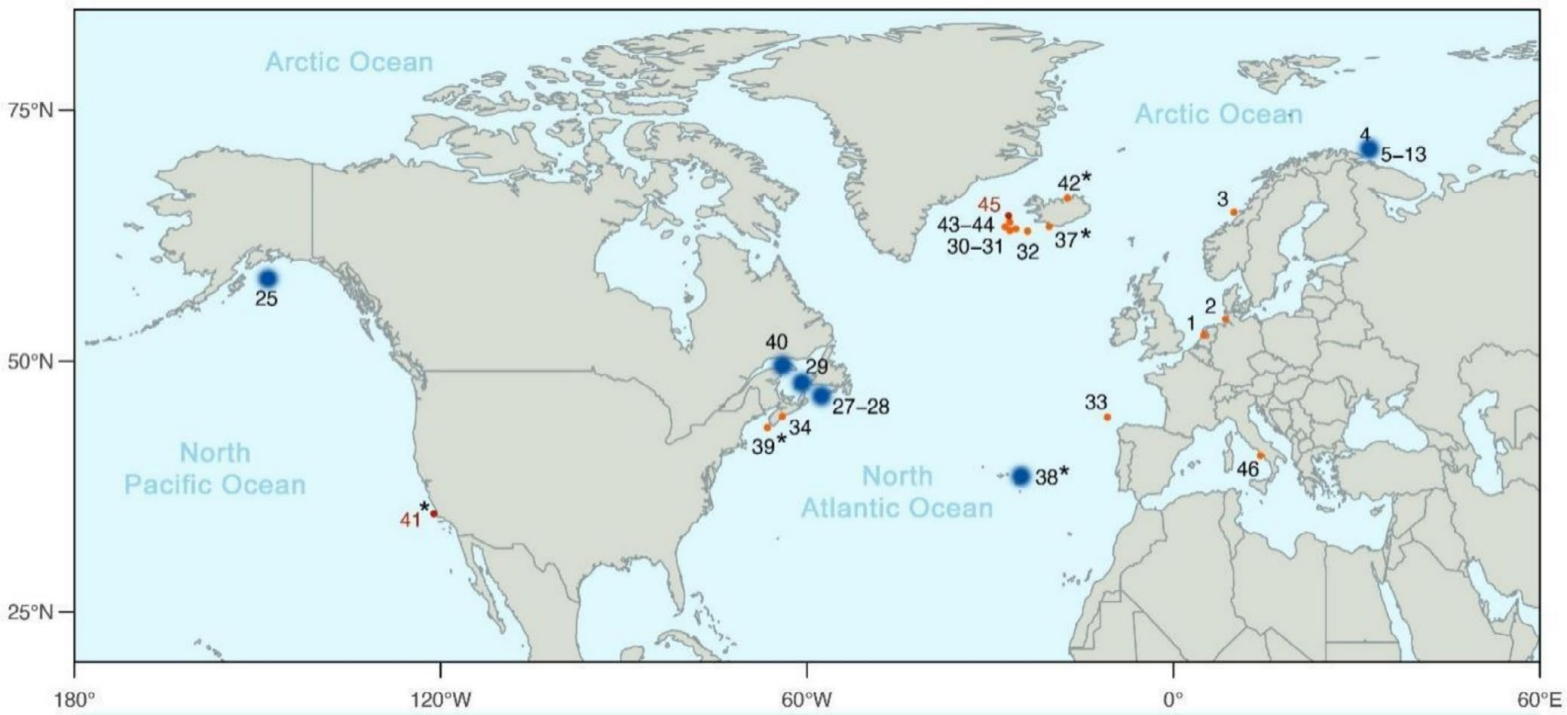
Geographical positions of blue‐fin whale hybrids across ocean basins. Numbers refer to reports summarised in Table 1 (Map no.). Large dots indicated hybrids for which location is not certain, while small red dots indicate hybrids for which location is known. Due to unknown locations, the following hybrids are not included: North Pacific (1932–1950), Northwest Atlantic (1966), Gulf of Alaska (1992–2004) and Antarctic (1995–1996). *Observations of hybrids alive (biopsy samples). Red numbers indicate second‐generation hybrids (41 and 45).

Even so, hybrids between blue and fin whales are surprising, because these two species diverged in the late Miocene, 10.5–7.5 Ma (Árnason et al. 2018; Wolf et al. 2023). Furthermore, both species have more closely related conspecifics: blue whales are most closely related to sei whales *B. borealis* and their close relatives (Bryde's *B. brydei* , Omura's *B. omurai* and Rice's *B. ricei* whales), while fin whales are most closely related to humpback whales *Megaptera novaeangliae* (Árnason et al. 2018; Wolf et al. 2023). Nevertheless, the presence of hybrids between both species has been documented for more than a century, dating back to the first records during modern whaling operations of the 1800s. In his reports, Cocks (1885, 1887) mentioned the capture of individuals called ‘bastards’ by whalers from the Lapland coast. Since then, alleged hybrids between blue and fin whales have been regularly reported across oceans, with many recently confirmed using various genetic methods (see Table 1 and Section 3). Most of the alleged and confirmed hybrids have been documented in the North Atlantic, but reports range widely over more than a century, off Norway, Iceland, eastern Canada, Alaska, Southern California, the Atlantic coasts of Spain, the Mediterranean Sea and even possibly Antarctica (Table 1, Figure 1). Here we synthesise available observations of blue‐fin hybrids across ocean basins from historical to recent records, highlighting those which have recently been genetically confirmed and providing information on morphological characteristics based on published and unpublished (Icelandic) information.

**TABLE 1:** Reporting of alleged and confirmed hybrids blue and fin whales across oceans.

Location	Dates	Map no.	Types of information	Sex	Mother species	Identification	References
Northeast Atlantic	1811, 1819, 1863	1–3	3 atypical whales	Unknown	Unknown	Morphology	Guldberg (1884)
Vardö, Norway	1885	4	Found dead, floating	Male	Unknown	Morphology	Cocks (1885)
Northeast Atlantic	1885–1886	5–13	9 (bastards) alleged hybrids caught	Unknown	Unknown	Morphology	Cocks (1887)
North Pacific	1932–1950	n.d.	11 alleged hybrids	Unknown	Unknown	Morphology	Zenkovich (1952)
Northeast of Kodiak Island USA	1965	25	Alleged hybrid	Unknown	Unknown	Morphology	Doroshenko (1970)
Northwest Atlantic	1966	n.d.	Alleged hybrid	Unknown	Unknown	Morphology	Mentioned in Bérubé and Aguilar (1998)
Newfoundland, Canada	1903	27–28	2 first generation hybrid	Unknown	Blue whale	Genetically confirmed	Jossey et al. (2024)
Newfoundland, Canada	1974	29	First generation hybrids	Unknown	Blue whale	Genetically confirmed	Jossey et al. (2024)
Iceland	1983	30	First generation hybrid	Male	Fin whale	Genetically confirmed	Árnason et al. (1991), Spilliaert et al. (1991)
Iceland	1986	31	First generation hybrid, carrying a foetus	Female	Blue whale	Genetically confirmed	Árnason et al. (1991), Spilliaert et al. (1991)
Iceland	1989	32	First generation hybrid	Male	Blue whale	Genetically confirmed	Árnason et al. (1991)
Spain	1984	33	First generation hybrid	Female	Blue whale	Genetically confirmed	Bérubé and Aguilar (1998)
Gulf of Maine	1992	34	Anomalous whale‐Alleged hybrid	Unknown	Unknown	Morphology	Bérubé and Aguilar (1998)
Gulf of Alaska	1992–2004	n.d.	Alleged hybrid	Male?	Unknown	Unique song track	Watkins et al. (2004), Stafford et al. (2007)
Antarctic	1995–1996	n.d.	Anomalous whale‐Alleged hybrid	Unknown	Unknown	Morphology	Mentioned in Bérubé and Aguilar (1998)
Iceland	1998	37	Anomalous alive whale	Unknown	Blue whale^a^	Genetically confirmed	Bérubé et al. (2017)
Azores, Portugal	Unknown	38	Alive hybrid	Unknown	Unknown	Genetically confirmed	Bérubé et al. (2017)
Gulf of Maine, Canada	Unknown	39	Alive hybrid	Unknown	Unknown	Genetically confirmed	Bérubé et al. (2017)
Gulf of St Lawrence, Canada	2002	40	First generation hybrid	Male	Fin whale	Genetically confirmed	Stacey (2022)
Northeast Pacific	2004	41	Second‐generation hybrid?	Male	Hybrid?	Genetically confirmed	Jefferson et al. (2021)
Iceland	2012	42	Alive‐first generation hybrid	Male	Blue whale	Genetically confirmed	Pampoulie et al. (2021)
Iceland	2013	43	First generation hybrid	Female	Blue whale	Genetically confirmed	Pampoulie et al. (2021)
Iceland	2018	44	First generation hybrid	Male	Blue whale	Genetically confirmed	Pampoulie et al. (2021)
Iceland	2018	45	Second‐generation hybrid	Male	Hybrid female	Genetically confirmed	Pampoulie et al. (2021)
Mediterranean Sea	2020	46	First generation hybrid	Female	Blue whale	Genetically confirmed	Fioravanti et al. (2022)

Abbreviation: n.d., not displayed on the map.

^a^ Martine Bérubé, personal communication.

## 2 Historical Observations

The earliest historical documentation of hybrids between blue and fin whales originates from reports of whaling operators from the 1800s, describing whales displaying morphological characteristics of both species (Cocks 1885, 1887). After the moratorium on commercial whaling, which became effective almost 100 years later, hybrid reports mainly came from sighting surveys or genetic analysis from biopsies, catches or strandings. Combining all these observations, the documentation of the first hybrid was in 1811 (Guldberg 1884) and the most recent one was in 2020 (Fioravanti et al. 2022).

The first report of hybrids has often been attributed to Cocks (Cocks 1885, 1887), who described the finding of a dead floating whale reported as a ‘bastard’ by whalers at Vardö, Norway (Cocks 1885), and the harvest of nine anomalous whales caught during whaling operations along the Lapland coast, Norway (Cocks 1887). These whales exhibited intermediate morphological traits, prompting speculation about hybridization long before genetic methods were available. Guldberg (1884) also mentioned three whales with abnormal characteristics that might have been hybrids. The first individual, caught in 1811, was approximately 32 ft long (9.75 m), and its skeleton was conserved at the Royal Museum of Leyde in the Netherlands. The second individual was described in 1819 and was a female about the same size, which stranded along the Holstein coast. Its skeleton was conserved in the Museum of Berlin. This individual was described as *B. rostrata* and drawn by Rudolphi, while Cuvier drew its skull under the name of Rorqual of the North (mentioned in Guldberg 1884). The skeleton of the third individual, apparently stranded in 1863 along the Norwegian coast, was conserved in the Museum of Bergen. While these three individuals were apparently used to describe a new species, the validation of this species remained challenging because of the lack of information. Guldberg (1884) stated that these individuals usually exhibited the shape and the colour pattern of the blue whale, but had intermediate characteristics between blue and fin whales in both their skull and skeleton. Given the limited morphological data and the confusion of whale identification until the late 1800s, Guldberg (1884) suggested that these individuals might have been hybrids between blue and fin whales rather than a distinct species.

One of these early reports of alleged hybrids, which is difficult to ascertain, is the report of Zenkovich (1952), who described the capture of 11 anomalous whales in the North Pacific by Russian whaling operations from 1932 to 1950. Doroshenko (1970) also reported the capture of a female in 1965 along the coast of Kodiak Island, USA, bearing the characteristics of both blue and fin whales, and exhibiting atypical colour patterns. The head was darker than the back and its right side was lighter than the left. The throat displayed small white spots which turned into white strips towards the flippers. The underside was light grey with numerous white spots. It also exhibited morphological characters like fin whales, such as a taller dorsal fin than that of blue whales.

Additional cases emerged from North Atlantic catches. Bérubé and Aguilar (1998) mentioned the processing of a 21.3 m whale, which was caught in the summer of 1966 at the whaling station at Blandford, Nova Scotia, Canada, that displayed abnormal characteristics. It was initially thought to be a hybrid between blue and fin whales due to the black colouration of its baleen plates and the symmetrical pigmentation of its body. Ultimately, it was recorded as a blue whale in the catches by the whaling inspector, but as a fin whale by the whaling factory. Bérubé and Aguilar (1998) also reported the sighting of an anomalous whale observed during a 1992 research cruise in the Gulf of Maine, and another one with an intermediate colouration and body shape between the blue and fin whales, which was sighted during a winter Antarctic research cruise in 1995–1996.

While these historical observations cannot be genetically verified, they provide suggestive evidence that hybridization between blue and fin whales has occurred for at least two centuries. The morphological descriptions, particularly the intermediate skull structure, atypical dorsal fin shape and mixed pigmentation, remain consistent with traits later confirmed in genetically identified hybrids. Most of the reported hybrids after these dates have been identified genetically, either from samples collected by whaling operations or genetic analyses of biopsies of anomalous whales (Table 1), apart from one individual, yet to be sighted, in the North Pacific, which was suggested to be a hybrid based on its unique song track (Watkins et al. 2004; Stafford et al. 2007).

## 3 Contemporary Observations: Genetic Confirmation of Hybrids

The advent of molecular techniques in the early 1990s enabled the first genetic confirmation of blue‐fin whale hybrids. Árnason et al. (1991) and Spilliaert et al. (1991) used restriction enzyme polymorphisms to confirm the identity of alleged hybrids captured during commercial catches in Icelandic waters in 1983, 1986 and 1989. These molecular approaches confirmed the status of these whales as hybrids, with two of them resulting from the mating of a blue whale mother and a fin whale father (1986, 1989), while the third (1983) had reversed parentage. Notably, the 1986 hybrid was a pregnant female, providing the first evidence of the fertility of hybrid whales. The fetus was concluded to have a blue whale father and a hybrid mother.

Several years later, using a 299 bp sequence from the control region of the mitochondrial DNA and α‐lactalbumin nucleotide sequences, Bérubé and Aguilar (1998) confirmed the presence of a hybrid caught off Caneliñas, Northwestern Spain, in 1984, resulting from the mating of a blue whale mother and a fin whale father. In 1998, a whale with a strange colouration and body shape was observed in Iceland, and a biopsy sample confirmed this individual as a hybrid between blue and fin whales (Bérubé et al. 2017). These observations were followed by a study on Icelandic individuals based on 24 microsatellite loci and the control region of the mitochondrial DNA (mtDNA) by Pampoulie et al. (2021), who genotyped five alleged hybrids for which samples were available from whaling operations in Iceland, and one biopsy sample of an alleged living hybrid observed in the north of the country since 2012. Two of these hybrids had already been genetically confirmed (Árnason et al. 1991; Spilliaert et al. 1991), while three additional samples had been collected during more recent whaling operations (one in 2013 and two in 2018). Of these six alleged hybrids, five were proven to be first generation resulting from the mating of a blue whale mother and a fin whale father. However, the sixth sample collected in 2018 (H2018‐2) exhibited higher assignment values to fin whales (circa 70%), consistent with a second‐generation hybrid resulting from a backcross with a male fin whale. The mother was then concluded to be a first‐generation hybrid. This was the first confirmed case of a living second‐generation hybrid of blue and fin whale.

Similar findings emerged from the eastern North Pacific. Jefferson et al. (2021) reported the presence of an alleged hybrid swimming along the southern coast of California, which was first observed in 2004, and repeatedly re‐sighted until 2020. This extended period of observation also allowed Jefferson et al. (2021) to document movements between wintering and summering grounds around the Gulf of California, Mexico and southern California, USA. The genetic analyses of a skin sample from this whale, based on 17 microsatellite loci, confirmed its hybrid status, with inferred ancestry of 68.5% to fin whale and 31.5% to blue whale. The authors suggested that this hybrid resulted from the mating of a blue whale mother and a fin whale father. However, the inferred ancestry for this hybrid strikingly resembled the observed values for the second‐generation hybrid caught in Iceland. While not explicitly suggesting it, Jefferson et al. (2021) probably identified the second second‐generation hybrid in the world, implying that the mother was a hybrid. This, together with the findings in Iceland, confirms that hybrid females can successfully mate with fin whale males and produce viable offspring.

Recent whole‐genome sequencing studies have also identified historical hybrids. In the North Atlantic, Jossey et al. (2024) analysed historical samples of blue whales to assess the hunting effect on the genetic diversity of the species. During their analyses, they noted high values of D‐statistics (a simple and powerful test for evaluating deviations from a strict bifurcating evolutionary history) for three individuals, two collected from 1903 (NWa5 and NWa6) and one from 1974 (NWa‐CM1), which suggests that these individuals were hybrids. Since none of them bore mtDNA from fin whales, the authors suggested that these hybrids were the result of matings from blue whale mothers and fin whale fathers.

In the Gulf of Saint‐Lawrence, in the North Atlantic Ocean, a sample collected from an identified blue whale included fin whale mtDNA and was therefore suggested to come from a hybrid resulting from the mating of a blue whale father and a fin whale mother (Stacey 2022). However, this hybrid could also have been a second‐generation hybrid with a fin whale mtDNA haplotype.

Fioravanti et al. (2022) investigated the origin of three fin whales stranded along the coasts of the Tyrrhenian Sea (Mediterranean Sea, Italy). Using the control region of the mitochondrial DNA and α‐lactalbumin (α‐lac) nuclear gene, they demonstrated that the mtDNA sequence of only two of the stranded whales could be attributed to fin whales, while the third sequence was attributed to a blue whale (ID531). The individual ID531 exhibited similar morphological characteristics to fin whales, and Fioravanti et al. (2022) therefore suggested that this individual was a hybrid. The mtDNA sequence of ID531 was identical to previously published sequences of two blue whales (14–99 and LK‐BM01‐2015) and a blue‐fin whale hybrid (H2018‐2) (Pampoulie et al. 2021), again consistent with blue whale maternal origin. The analysis of the α‐lactalbumin nuclear gene confirmed the hybrid status of ID531, indicating a successful mating of a mother blue whale and a father fin whale. Since this was a stranded animal found in a hidden inlet at Cala del Rio in Capri, Naples, Italy, on November 7, 2020, this was the first hybrid record in the Mediterranean Sea.

So far, 16 out of 17 (94%) of the genetic identification of hybrids between blue and fin whales were from the North Atlantic Ocean and the Mediterranean Sea, with the sole exception coming from southern California. Collectively, these findings demonstrate that hybridization between blue and fin whales is not only ongoing but also capable of producing fertile individuals and, consequently, second‐generation hybrids. Across ocean basins, the pattern remains strongly unidirectional: in 11 of 13 confirmed first‐generation cases (excluding Stacey 2022), hybrids resulted from a blue whale mother and a fin whale father (see Table 1). There is only a 0.011 probability of this occurring by chance (assuming a binomial distribution with 50% probability of a fin whale father).

## 4 Morphological Features of Hybrids Compared With Their Blue and Fin Whale Parental Species

**FIGURE 2:**
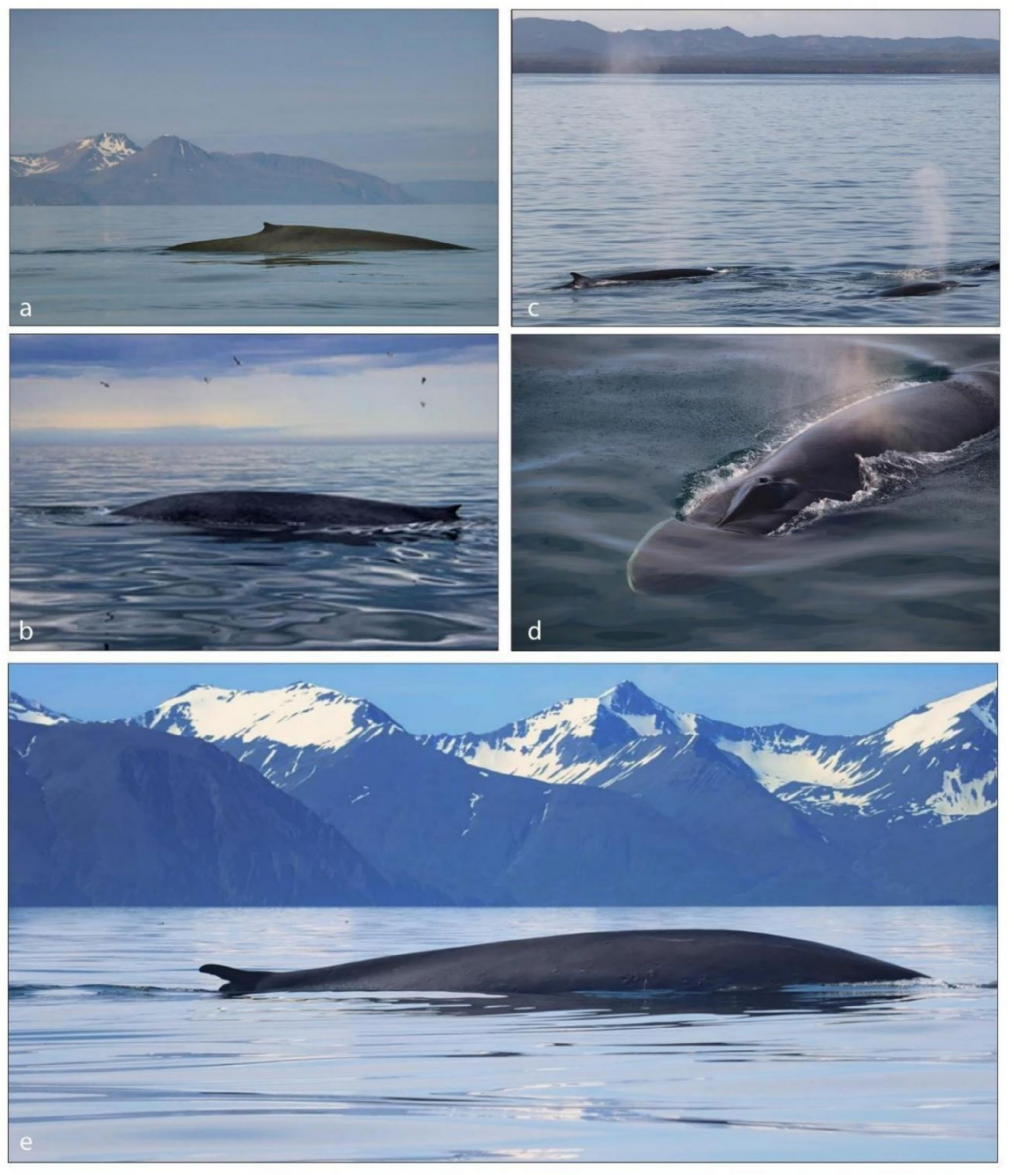
Blue whale (a, b), fin whale (c, d) and a hybrid (e) in Icelandic waters. Pictures were kindly provided by Gentle Giants, Húsavík (a, b, e; website: https://www.gentlegiants.is) and Special Tours Iceland (c, d; website: https://specialtours.is). (e) The well‐known hybrid swimming in the water North of Skjálfandi bay in Iceland since 2012 (reported as HALIVE in Pampoulie et al. 2021) is a male hybrid, resulting from the successful mating of a female blue whale and a male fin whale. It is usually seen within a group of blue whales.

The fin whale, with a body colour varying from light to dark grey (Figure 2c,d), is one of the few baleen whales to display asymmetrical coloration and patterns. While its left anterior body side is dark, the right one is pigmented with a lighter coloration. Similarly, the left side of the head tends to be dark grey while the right side usually displays a range of light and dark colouration patterns.

**FIGURE 3:**
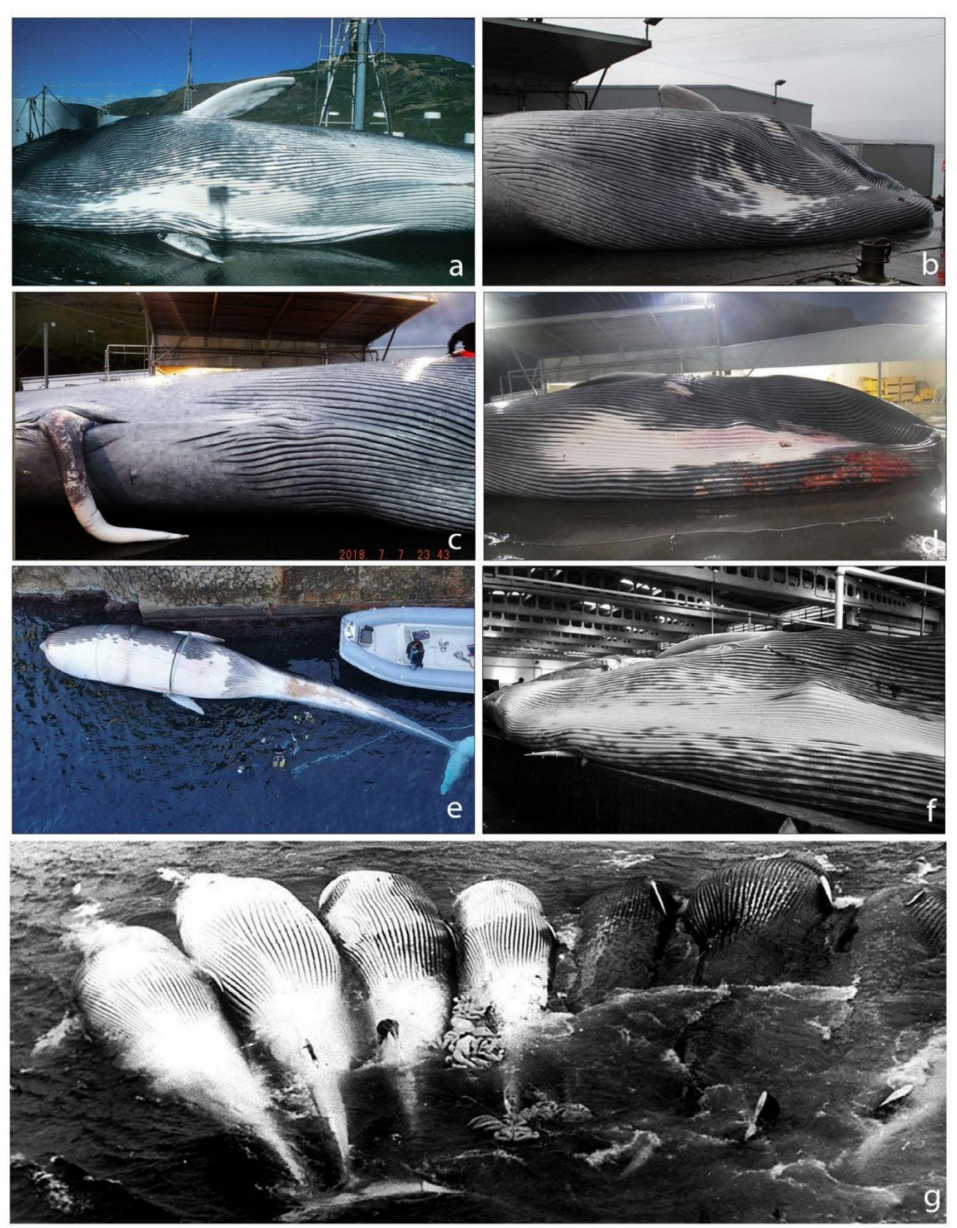
Ventral sides of (a, b, c) the first‐generation hybrids caught in Icelandic waters in 1989, 2013 and 2018 (reported respectively as H1989, H2013 and H2018‐1 in Pampoulie et al. 2021), (d) the second‐generation hybrid caught in Icelandic waters in 2018 (reported as H2018‐2 in Pampoulie et al. 2021), (e) the hybrid ID531 stranded in hidden inlet, at Cala del Rio (Capri, Naples, Italy), on November 7th, 2020 (Fioravanti et al. 2022), (f) the hybrid caught in northwestern Spain in 1984 (Bérubé and Aguilar 1998) and (g) fin (4 individuals on the left) and blue whales (3 individuals on the right) caught during the Antarctic whaling season 1951/2–1952/3 (Permission and rights received from PolarJournal.com, photographer Cornelius Gransbergen).

A distinctive feature of the fin whale right side is the white patch present on the mandible and the top and right side of the snout, as well as the white/yellowish baleen plates covering the anterior third of the mandible total length (True 1904; Allen 1916; Agler et al. 1990; Tershy and Wiley 1992) (Figures 2d and 5g). The ventral side of fin whales is usually white or light grey but can exhibit a large variation in the extent of white (Figure 3g). The dorsal fin, which is placed at the fourth posterior end of the body, is falcate (hook‐shaped) and of intermediate size among balaenopterids (Figure 2c). Of note is also the presence of a white central ‘V’ shaped chevron posterior to the blowhole, curving on the side of the head, associated with a unique set of white‐greyish stripes. Asymmetrical patterns and white‐greyish chevrons are visible on the dorsal side (Agler et al. 1990, 1992).

**FIGURE 4:**
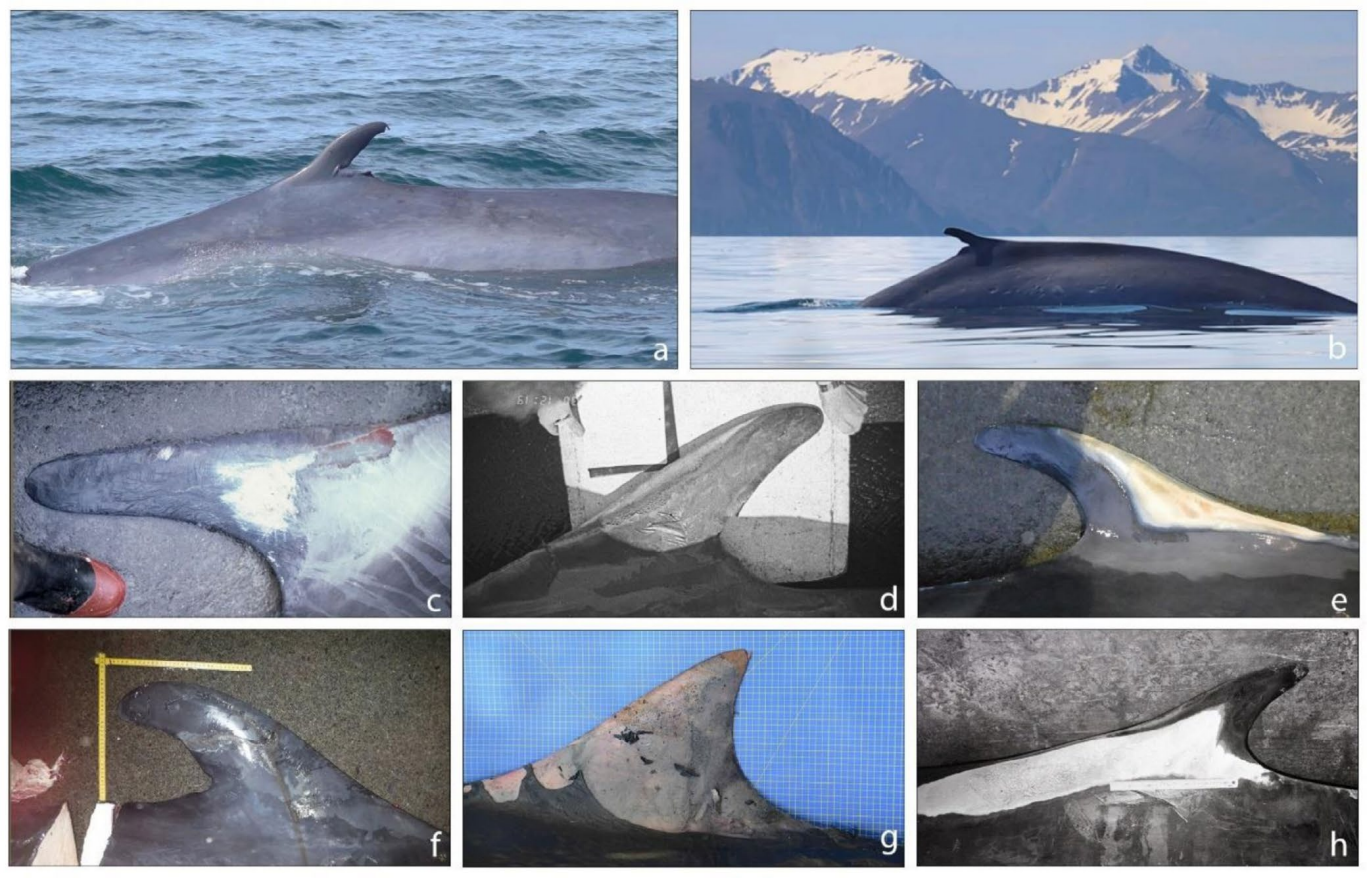
Dorsal fins of (a) the genetically confirmed hybrid regularly observed in Southern California from 2004 to 2020 (Jefferson et al. 2021; Photo courtesy of Thomas Jefferson); (b) the well‐known hybrid swimming in the water north of Skjálfandi bay in Iceland since 2012 (reported as HALIVE in Pampoulie et al. 2021; Photo courtesy of Gentle Giants Húsavík, website: httpS://www.gentlegiants.is); (c, d, e) the first‐generation hybrids caught in Icelandic water in 1986, 1989 and 2018 (reported respectively as H1986, H1989 and H2018‐1 in Pampoulie et al. 2021); (f) the second‐generation hybrid caught in Icelandic waters in 2018 (reported as H2018‐2 in Pampoulie et al. 2021); (g) the hybrid ID531 stranded in hidden inlet, at Cala del Rio (Capri, Naples, Italy; Photo courtesy of Dr. Caputo Barucchi) in 2020 (Fioravanti et al. 2022), and (h) the hybrid caught in northwestern Spain in 1984 (Bérubé and Aguilar 1998).

The blue whale exhibits fewer diagnostic morphological features. It has a light blue‐greyish colouration all over the body (Figure 2a,b), with a light blue and characteristic mottled pattern which can appear blue from the sky or under water. The ventral side is characteristically darker grey and not white (Figure 3g). The throat is light/grey blue and usually not mottled like the rest of the body. The dorsal fin is small and triangular, and positioned in the last fourth of the body, which is further back than in any other whale species. It can have a variety of different shapes but is seldom falcate and is much smaller than in fin whales (mean height 0.27 m vs. 0.47 m in the Southern Hemisphere, Mackintosh and Wheeler 1929). The underside of the pectoral fins is typically white (Figure 3g), and the baleen plates are black.

Historical and recent descriptions suggest that blue‐fin hybrids usually exhibit morphological characteristics intermediate to both species. Cocks (1885, 1887) mentioned large whales described as grey rather than white on the ventral side but with partially white baleen plates on the right side, while Guldberg (1884) mentioned whales with the appearance and colours of the blue whale but with an intermediate number of vertebrae and an intermediate shape and size of the skull between the blue and the fin whale. Bérubé and Aguilar (1998) estimated the Caneliñas hybrid to be 4 year old based on earplugs, and it was significantly longer (19.4 m vs. 17.4 m, *p* < 0.01) than 21 fin whales of the same age inhabiting these waters. Furthermore, fin whales at that length should have been sexually mature, but this hybrid was sexually immature as it did not present any ovulation corpora in the ovaries.

**FIGURE 5:**
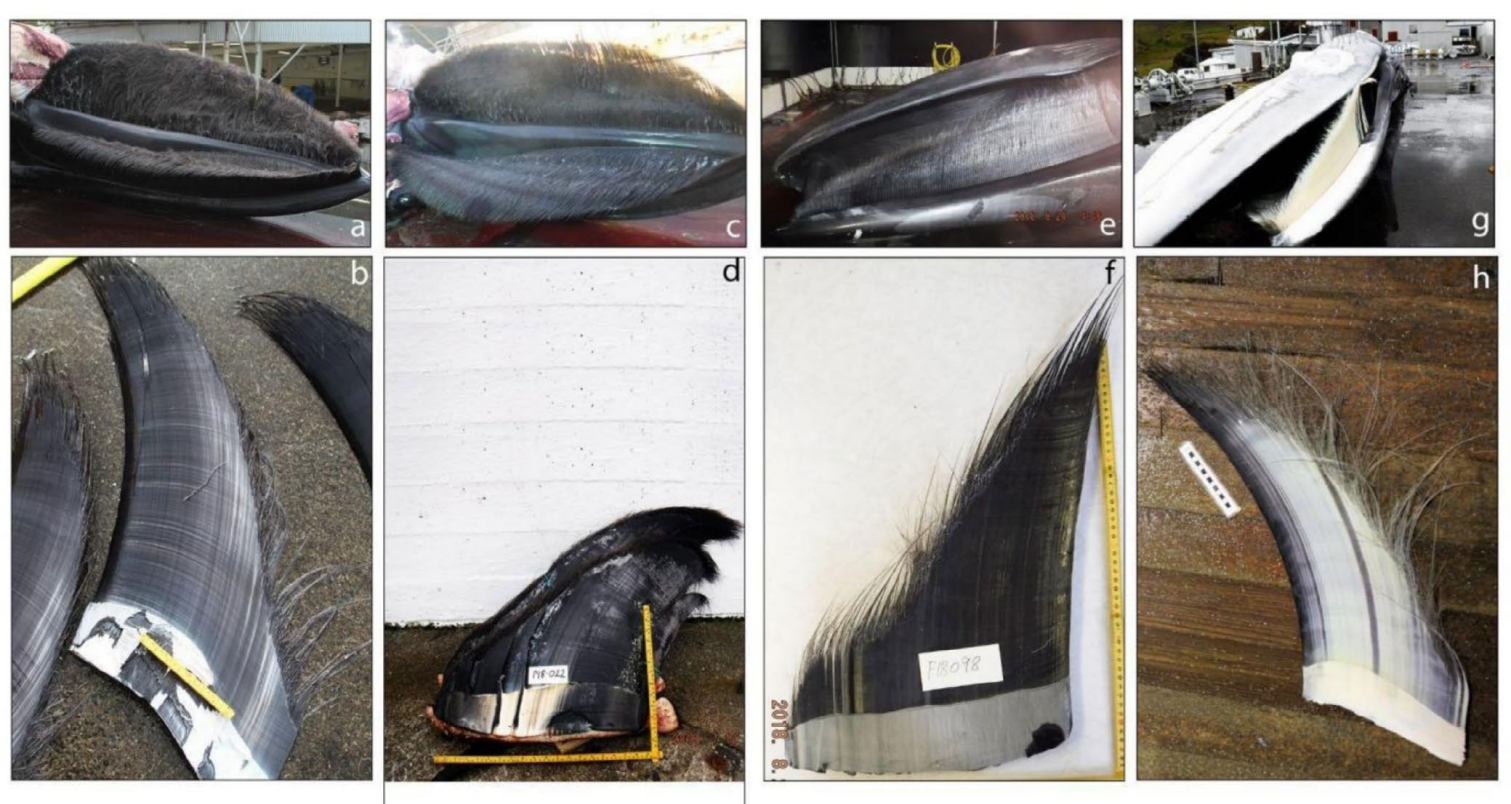
Pictures of the mouth and baleen plates of hybrid whales (a, b, c, d, e, f) and a fin whale (g, h) caught in Icelandic waters. (a, b) first generation hybrid caught in 2013; (c, d) first generation hybrid caught in 2018 (H2018‐1); (e, f) second‐generation hybrid caught in 2018 (H2018‐2); (g, h) fin whale caught in 2022.

While historical observations often mentioned very few morphological characteristics, recent observations of hybrids from catches include more complete descriptions (Figures 3, 4, and 5).

Apart from the first‐generation hybrid caught in Iceland in 2018 (Pampoulie et al. 2021; Figure 3c), which displays a ventral light/grey side, most of the hybrids caught during whaling operations presented a dark ventral side split by an asymmetrical white patch of various sizes and shapes, often also surrounded by white spots of various sizes (Figure 3a,b,d,f). This patch of white colouration on the ventral side is smaller than the typical white pattern in fin whales (Figure 3g). Also, in the hybrids, the ventral side of the caudal fin was dark, with some clearer patches near the shaft, while it is white with grey‐slate borders in fin whales and is uniformly grey in blue whales.

One of the striking hybrid characteristics is their dorsal fin, which sometimes has an atypical, pronounced hooked shape with abnormalities and is usually comparable in size to that of fin whales and taller than that of blue whales. However, caution should be taken when considering this trait since most dorsal fin observations come from hybrids caught during whaling operations targeting fin whales, for example, individuals most likely to be exhibiting fin‐whale‐like dorsal fin characteristics (Figure 4). Few pictures of dorsal fins of living hybrids are available (Figure 4), with the only documented cases coming from two individuals: one observed in Iceland (HALIVE in Pampoulie et al. 2021) and one in Southern California (Jefferson et al. 2021).

Both animals had a high dorsal fin compared to their parent species, with very similar abnormalities (see Figure 4a,b). While Jefferson et al. (2021) suggested that this dorsal fin abnormality might reflect damage due to entanglement with fishing gear or vessel strike, it is surprising to observe an almost identical and highly anomalous dorsal fin pattern for two hybrids located in different ocean basins. In general, most of the pictures of dorsal fin available clearly show a distinctive pattern for the hybrids, that is, their dorsal fins are usually taller than those of blue whales and have a very specific tall hooked‐shape form (Figure 4).

Except for the first‐generation hybrid caught in the Mediterranean Sea (Fioravanti et al. 2022), baleen plates of hybrids are usually black on both sides of the mouth (Figure 5) but often display paler vertical strips (Figure 5b,d,f). The characteristic white/yellowish baleen plates on the right side of the mouth, typical of fin whales (Figure 5g,h), are absent.

Morphological characteristics of hybrids also undoubtedly depend on the level of hybridization. One of the best morphologically described hybrids is the hybrid alive observed in Southern California (Jefferson et al. 2021), which might be a second‐generation hybrid based on the ancestry inference proportion (68.5% to fin whale). Its dorsal fin exhibits a tall, hooked‐shape form atypical for fin whales; it has light white chevrons between the flippers, a snout and a rounded upper rostrum like fin whales and also exhibits colouration patterns intermediate to blue and fin whales. The white colouration on the right mandible is absent.

## 5 Unidirectionality of the Hybridization and Second‐Generation Hybrids

With the advances of genetic methods in the last decades, the information retrieved on hybrids has shed light on many aspects of their biology, which still need confirmation (see Table 1). For many years, hybrids were thought to be infertile (particularly for males), but this was overturned by a pregnant female hybrid that was caught in Icelandic waters (Spilliaert et al. 1991) and recent genetically confirmed second‐generation hybrids (Jefferson et al. 2021; Pampoulie et al. 2021). Genome analyses conducted in the North Atlantic have suggested that gene flow seemed to be unidirectional from fin to blue whale, the latter displaying *circa* 3.5% of fin whale genetic material (Jossey et al. 2024). These results nevertheless depict ancestral hybridization processes, since no apparent recent introgressive signals were detected between both animals despite known hybridisation for more than a century (Westbury et al. 2019). Moreover, the recent identification of a large number of hybrids across oceans and the discovery of second‐generation hybrids (Jefferson et al. 2021; Pampoulie et al. 2021) combined with evidence of unidirectional hybridisation (Bérubé and Aguilar 1998; Jefferson et al. 2021; Pampoulie et al. 2021; Fioravanti et al. 2022; Jossey et al. 2024; Table 1) seem to contradict the genomic studies and are alarming signs for blue whale recovery. Genetic confirmation that the hybrids were the result of a male fin whale siring a female blue whale in 11 cases out of 13 (Table 1) might reflect the inherent difficulty of female blue whales to find a conspecific mate (Pampoulie et al. 2021), following the predictions of the sexual selection hypothesis for unidirectional hybridization (Wirtz 1999). The two second‐generation hybrids documented so far, one in Iceland and one likely in the eastern North Pacific, both exhibited a higher percentage of inferred ancestry to fin whale (*circa* 70%), which tends to indicate that the current hybridization process leads to the introgression of blue whale genetic material into the fin whale's genome. While most baleen whale species have recovered from historical whaling, blue whales were more heavily depleted, and some populations have not recovered (Thomas et al. 2016). Today, fin whales are far more abundant than blue whales in almost all oceans, especially in the North Atlantic, where fin whales are 25 times more abundant (Pike et al. 2019). This, and the current knowledge on hybridization suggests that the directionality in the hybridization might lead to a reproductive output loss for blue whales in the North Atlantic, and perhaps also worldwide, a hypothesis that is worrying given that most populations of this species are still depleted. The effect of this depletion has been demonstrated by genomic studies, which detected substantial levels of inbreeding signals, evidenced by frequent and extended runs of homozygosity (Wolf et al. 2023). In addition to the signal of inbreeding and the potential effect of unidirectional hybridization, the blue whale is a stenophagous feeder specialised in krill species, which makes it particularly sensitive to climate‐driven variability in prey distribution and abundance (Jolliffe et al. 2026). The combination of all these factors might explain the slow recovery of blue whale populations across ocean basins compared to other baleen whales.

The frequency of hybrids between blue and fin whales is also difficult to estimate, and it would therefore be crucial to register and monitor the presence of suspicious whales exhibiting characteristics of both species and, whenever possible, to obtain biopsy or sloughed skin samples. However, the hypothesis of unidirectional hybridization needs confirmation since all but five of the genetically confirmed hybrids came from commercial operations targeting fin whales (i.e., individual whales therefore resembling fin whales), the exceptions being hybrids from the Northeast Pacific (Jefferson et al. 2021), Newfoundland (Jossey et al. 2024), Iceland (living hybrids from 1998 and 2012) and the Mediterranean Sea (Fioravanti et al. 2022).

## 6 Future Perspectives

Hybridization is generally considered to be more common among species with recent evolutionary divergence, typically within the same phylogenetic group (Mallet 2007). The occurrence of hybrids between blue and fin whales is therefore puzzling, since these species diverged in the late Miocene, approximately 8–10 million years ago (Árnason et al. 2018) and are regarded as distinct conspecifics (Árnason et al. 2018; Westbury et al. 2019; Wolf et al. 2023). Within cetaceans, only one other case of hybrids with back‐cross has been confirmed in the Balaenopteridae family: the hybrids detected between the North Atlantic minke whale ( *B. acutorostrata* ) and the Antarctic minke whale ( *B. bonaerensis* ) (Glover et al. 2013).

Advances in genetic techniques have improved our understanding of parental contributions to hybrid genomes, yet the implications for both species require further investigation using new available genomic resources. Indeed, the comparative genomic studies performed on blue and fin whales so far, including the detection of introgression, were mainly based on the utilisation of the blue whale genome as a reference, which can lead to inaccurate genetic estimates such as ‘mapping depth and mapping quality, as well as core population genetic estimates, such as heterozygosity levels, nucleotide diversity (*π*), and cross‐species genetic divergence (DXY)’ (Maurstad et al. 2025). This could have led to imprecise estimates of time of divergence and an incorrect signal of introgressions. In addition, hybrids were most of the time not included in such studies. The genome of both species is now available (Wolf et al. 2022, Bukhman et al. 2024, Wolf et al. 2025, Davison and Morin 2026), and a re‐evaluation and replication of earlier introgression analyses using these new expanded genomic resources is therefore necessary. Ultimately, a full genome analysis of both parental species together with hybrids will uncover the genomic implications for both species and fully fathom the direction of introgression.

Given existing data, most confirmed hybrids result from mating between a blue whale mother and a fin whale father, though the underlying causes remain unclear. The literature on the potential causes of hybridization among cetaceans, and especially blue and fin whales, remains limited. However, Crossman et al. (2016) suggested that cetacean species had a high probability of producing hybrids when morphological and behavioural traits such as vocalisation frequency and body size were similar between the two potential parental species. Regardless of the mechanism, hybridization between blue and fin whales has been proven to persist for over two centuries and has likely influenced the genomic architecture of both species. Current genetic studies on hybrids suggests introgression primarily from blue to fin whales, although genomic analyses indicate a more complex pattern due to the difficulty in distinguishing between historical and recent introgression, and possibly methodological concerns (Árnason et al. 2018; Westbury et al. 2019; Wolf et al. 2023). The identification of second‐generation hybrids and apparent unidirectional hybridization supports this trend but warrants confirmation. If these patterns hold across ocean basins, they may reduce blue whale reproductive output and facilitate introgression of blue whale genetic material into fin whale. Assessing the extent of hybridization and the genomic characteristics of hybrids is therefore critical. Monitoring individuals with atypical morphological traits and collecting biopsy or sloughed skin samples for genetic analysis will be essential to confirm hybrid status and evaluate the impact of hybridization on both species.

## Author Contributions

**Christophe Pampoulie:** conceptualization (lead), data curation (equal), investigation (equal), supervision (lead), validation (equal), visualization (equal), writing – original draft (lead), writing – review and editing (equal). **Valerie Chosson:** data curation (equal), validation (equal), visualization (equal), writing – review and editing (equal). **Sverrir Daníel Halldórsson:** validation (equal), visualization (equal), writing – review and editing (equal). **Trevor A. Branch:** investigation (equal), validation (equal), visualization (equal), writing – review and editing (equal). **Marc Ruiz‐Sagalés:** validation (equal), visualization (equal), writing – review and editing (equal). **Alex Aguilar:** investigation (equal), validation (equal), visualization (equal), writing – review and editing (equal). **Guðjón M. Sigurðsson:** validation (equal), visualization (equal), writing – review and editing (equal).

## Funding

The authors have nothing to report.

## Ethics Statement

The biological data used in the present review were collected during routine monitoring under the Marine and Freshwater Research Institute of Iceland.

## Conflicts of Interest

The authors declare no conflicts of interest.

## Data Availability

The authors have nothing to report.
